# Identifying risk patterns for sudden cardiac death in athletes: A clustering and principal component analysis approach

**DOI:** 10.1371/journal.pone.0339377

**Published:** 2026-01-14

**Authors:** Giacinto Angelo Sgarro, Paride Vasco, Domenico Santoro, Luca Grilli, Marco Giglio, Natale Daniele Brunetti, Luigi Traetta, Giuseppe Cibelli, Anna Antonia Valenzano

**Affiliations:** 1 Department of Social Sciences, University of Foggia, Foggia, FG, Italy; 2 Department of Clinical and Experimental Medicine, University of Foggia, Foggia, FG, Italy; 3 Department of Economics, Statistics and Business, Faculty of Technological and Innovation Sciences, Universitas Mercatorum, Rome, RM, Italy; 4 Department of Economics, University of Foggia, Foggia, FG, Italy; 5 Department of Medical and Surgical Sciences, University of Foggia, Foggia, FG, Italy; 6 Department of Humanities, Letters, Cultural Heritage and Educational Studies, University of Foggia, Foggia, FG, Italy; Cedars-Sinai Heart Institute, UNITED STATES OF AMERICA

## Abstract

Sudden Cardiac Death (SCD) is a critical and unexpected condition that occurs due to cardiac causes within one hour of the onset of acute cardiovascular symptoms or twenty-four hours in unwitnessed cases. Despite advancements in cardiovascular medicine, practical methods for predicting SCD are still lacking, and there are no standardized systems to identify individuals at risk, especially in seemingly healthy populations such as athletes. In this study, we employed hierarchical clustering and principal component analysis (PCA) on data from 711 competitive athletes, revealing distinct patterns and cluster distributions in PCA space. Specifically, Clustering revealed characteristic feature combinations associated with increased SCD risk in athletes. Notably, certain clusters shared traits, including participation in Class C sports, sinus tachycardia, ventricular pre-excitation, personal or family history of heart disease, T-wave inversions, and prolonged QTc intervals. PCA helped visualize these patterns in distinct spatial regions, highlighting underlying structures and aiding intuitive risk interpretation. These results enable scientists to derive cluster metrics that serve as reference points for classifying new individuals and visually representing risk patterns in a clear graphical format. These findings establish a foundation for predictive tools that, with additional clinical validation, could aid in the prevention of SCD. The dataset used in this study, along with the clustering and PCA results, is available to the scientific community in an open format, together with the necessary tools and scripts to enable independent experimentation and further analysis.

## Introduction

Regular physical activity is widely recognized for its numerous health benefits, including significant reductions in all-cause mortality and the incidence of cardiovascular diseases, cancer, and metabolic disorders [1–5]. Despite these advantages, the risk of SCD remains a challenging reality, even among healthy individuals and athletes. Sudden cardiac death is defined as an unexpected death caused by cardiac conditions occurring within one hour—or within twenty-four hours in unwitnessed cases—after the onset of acute symptoms, without external contributing factors [6]. In Western countries, SCD accounts for an estimated 13–20% of all deaths [6,7], with its occurrence influenced by age, gender, ethnicity, and the type of physical activity practiced.

### Incidence and prevalence

Age plays a crucial role in the etiology of SCD. In individuals under thirty-five, hereditary conditions such as cardiomyopathies and channelopathies are the predominant causes. Notably, hypertrophic cardiomyopathy is responsible for 36% of SCD cases in young athletes in the United States [8], whereas arrhythmogenic idiopathic ventricular cardiomyopathy accounts for 23% of cases in Italy, with hypertrophic cardiomyopathy contributing to only 2% [9]. Conversely, in adults over thirty-five, atherosclerotic coronary artery disease becomes the leading cause of SCD, comprising 73% of cases in military personnel within this age group [10]. The incidence of SCD also increases with age among athletes, rising from 0.47–1.21 per 100,000 person-years in young competitive athletes to 6.64 per 100,000 person-years in those over thirty-five [11].

### Risk factors and co-morbidities

Gender disparities are another critical aspect of SCD risk. Studies consistently reveal that male athletes face a higher risk compared to females. Finocchiaro et al. (2021) [12] reported an SCD incidence rate of 2.6 per 100,000 person-years in male athletes versus 1.1 per 100,000 in females [9]. Furthermore, a UK registry of 748 SCD cases during sports activities showed that only 13% involved women, with sudden deaths during intense physical exertion occurring significantly less frequently in females than in males (58% versus 83%) [12]. Possible explanations include gender differences in physiological cardiac adaptations to exercise, chamber remodeling, myocardial fibrosis prevalence, and estrogens’ protective role in women [12–14]. Ethnicity also shapes SCD risk. Young African American athletes experience a threefold higher incidence compared to their white counterparts, at 5.6 per 100,000 annually [8,15,16]. This aligns with studies from the United States highlighting racial disparities in SCD occurrence. The type and intensity of physical activity further modulate SCD risk. Competitive athletes, exposed to higher training loads and adrenergic surges, face more significant risks than recreational athletes, with SCD rates of 1 in 100,000 compared to 0.32 in 100,000, respectively [7]. Intense exercise may exacerbate pre-existing cardiac abnormalities through mechanisms such as dehydration, electrolyte imbalances, and acid-base disturbances, triggering fatal arrhythmias [8,17,18].

### Challenges in early detection and screening

Early detection remains challenging despite the apparent associations between SCD and these risk factors. Many SCD victims present no symptoms or experience nonspecific warning signs. Post-mortem examinations often reveal structurally normal hearts in younger individuals, suggesting undetectable electrical abnormalities as the underlying cause in 41% of cases [19]. When anomalies are detected, they may include idiopathic left ventricular hypertrophy or myocardial fibrosis, though their clinical significance remains debated [20]. In Italy, mandatory pre-participation physical evaluations (PPPE), including electrocardiograms (ECG), has significantly reduced the incidence of SCD linked to hypertrophic cardiomyopathy [9,21]. However, screening presents limitations: high costs, frequent false positives, and an inability to identify all at-risk individuals [22,23].

### Methodological and technological advances

Artificial intelligence (AI) has emerged as a promising tool for overcoming these limitations. By processing complex, heterogeneous data—clinical measurements, environmental observations, and experimental results—AI creates predictive models for human diseases. Machine learning algorithms have shown efficacy in forecasting hypertension and atrial fibrillation using datasets derived from ECG and electronic health records [24–26]. Advanced methodologies such as hierarchical clustering (HC) and PCA offer new avenues for SCD risk stratification. Hierarchical clustering identifies groups of patients with shared risk profiles by analyzing intrinsic data relationships. At the same time, PCA reduces data dimensionality, isolating key variables for more intuitive visualization of risk factors and patient clusters [27–30]. The present study aims to develop a novel SCD risk diagnostic system by applying a HC algorithm to a dataset collected from hospital patients. This method enables the targeted identification and stratification of risk, revealing clusters of individuals at risk. We propose a PCA-based visualization approach that translates complex patient data into graphical representations, highlighting risk profiles and interconnected factors. The clustering outcomes, visualized through PCA, depict patient risk stratification and are illustrated in Figs 4 and 5. In both images, the elements belonging to the blue cluster are considered non-risk subjects. Using these data and visualizations, it is also possible to classify and position new patients within the PCA space, as demonstrated in Figs 10 and 11. These methods can advance our understanding of SCD risk and support clinical decision-making through data-driven insights. S1 Fig presents a graphical representation of our work.

## Materials and methods

### Data collection and feature extraction

#### Data collection.

In this study, we utilized a dataset comprising 711 athletes, each represented by a row, along with 151 initial features derived from pre-participation physical evaluations (PPPEs) conducted at the Sports Medicine Unit of the Policlinics of Foggia between 2019 and 2024. We used Excel as our primary tool for data management, systematically organizing and categorizing the information. For each athlete, we collected demographic and personal data (age, date of birth, gender, ethnicity, sport discipline and category), family history of SCD and cardiovascular/metabolic diseases, and personal medical history including cardiac events and medication use. The medical history encompassed previous cardiac events or diagnoses, including hypertrophic cardiomyopathy, arrhythmogenic cardiomyopathy, and medication use, particularly those that affect heart function or electrolyte balance. We also documented any history of syncope, palpitations, or chest pain during exercise. Lifestyle and training data included the type and intensity of sports practiced, distinguishing between competitive and recreational activities, as well as the duration and frequency of training sessions.

#### Cardiac and functional assessments.

Cardiac assessments included ECG findings such as QRS complex abnormalities and T-wave inversions. Echocardiography provided measures of chamber size and myocardial thickness. Cardiac MRI was used to identify fibrosis or structural anomalies. Exercise stress tests evaluated exertion-induced arrhythmias. Genetic testing was conducted to screen for mutations associated with cardiomyopathies and channelopathies. Functional tests analyzed heart rate variability (HRV), blood pressure responses during and after exercise, and blood markers like troponin levels and electrolytes. Psychosocial factors were also assessed, including stress levels, coping mechanisms, sleep patterns, and overall quality of life. These examinations assessed body composition (BMI), resting blood pressure, musculoskeletal, respiratory, cardiovascular, and other systems. Additionally, mandatory tests, including electrocardiograms (ECGs) at rest and under exertion, were performed to identify potential abnormalities.

#### Feature selection and dataset creation.

At the outset, the dataset included a total of 151 variables, encompassing a broad range of demographic, clinical, and instrumental features. These are fully listed and described in Table 13). To enable a more interpretable and targeted analysis, we organized the variables into three hierarchical subsets. The first subset consists of 45 features, defined as the “Literature + Extra” group, which includes variables supported by both established research and emerging clinical hypotheses. The second subset, referred to as “Complete Literature,” narrows this down to 26 features that are strongly corroborated by evidence in the scientific literature regarding sudden cardiac death (SCD). Finally, from this set of 26, we performed a further selection of the 8 variables most strongly correlated with SCD, based on statistical relationships and clinical relevance, referred to as “Literature” group. All three groups—45, 26, and 8 features—are described and numerically identified in Table 14.

For the purpose of this study, we chose to work with the datasets corresponding to the “Literature” and “Literature + Extra” groups, respectively **dataset_L_** and **dataset_LE_**.

The selection criteria used to build **dataset_L_** were informed by the meta-analysis conducted by Harmon et al. [31], which examined the effectiveness of screening history, physical examinations, and ECGs in detecting potentially fatal cardiac disorders in athletes. This study analyzed 47,137 athletes and reported a prevalence of 160 severe cardiovascular conditions, which translates to 0.3%. The meta-analysis highlighted several key disorders associated with SCD, such as Wolff-Parkinson-White syndrome (WPW), long QT syndrome (LQTS), hypertrophic cardiomyopathy (HCM), dilated cardiomyopathy, coronary artery disease (CAD), arrhythmogenic right ventricular cardiomyopathy (ARVC), myocarditis, and Brugada syndrome. Guided by these findings, we carefully selected variables that showed strong correlations with SCD risk. The features included in **dataset_L_** consisted of ECG findings like T-wave inversion, short PR intervals, Q waves, ST-segment depression, ventricular extrasystoles, and Brugada type 1 pattern. We also incorporated clinical indicators such as family history of CAD and personal history of myocardial infarction. To further account for physiological risk factors, we added metrics like body mass index (BMI) and blood pressure, which are closely linked to left ventricular hypertrophy and other cardiovascular abnormalities associated with SCD. In parallel, we constructed **dataset_LE_**, extending the analysis by combining the full set of 26 literature-based features with additional variables derived from exploratory clinical observations and emerging hypotheses. This resulted in a broader feature space comprising 45 variables.

#### Data analysis.

We applied clustering analyses to both datasets to identify groups of athletes with shared characteristics, and used PCA for dimensionality reduction and pattern recognition. Comparing results allowed us to evaluate the impact of additional variables on clustering and SCD risk assessment.

The complete forty-five-feature dataset is available for download at https://github.com/hyacintus/Sudden-Cardiac-Death-Survey.git

Alongside the dataset, the repository also provides the tools and scripts necessary to reproduce the research results, perform testing, and independently evaluate the system. Further in the article, links to specific subfolders within the repository are provided in the relevant sections for ease of access.

### Hierarchical clustering for pattern recognition

We utilized a hierarchical clustering algorithm to categorize athletes based on their risk of SCD to identify distinct risk profiles. This methodology was organized into four phases: dataset preparation, clustering, dendrogram analysis and cutting, and analysis of clusters and prototypes.

#### Dataset preparation.

We prepared two datasets of 711 athletes by selecting features associated with SCD risk as identified in existing literature. These features included eight or forty-five variables related to risk factors, physiological parameters, and resting and stress ECG features. The majority of variables are binary (Yes/No), except Body Mass Index (BMI) and Heart Rate, which are continuous (see Table 14 in Appendix section).

To ensure consistency and allow meaningful comparisons between features during mathematical modeling and clustering, we applied min-max normalization to the continuous variables (BMI and Heart Rate). In datasets containing variables with different value ranges, features with wider or numerically larger scales can dominate the outcome of algorithms, skewing the results and reducing interpretability. Normalization addresses this issue by rescaling all features to the same range, allowing them to contribute equally to the analysis.

In our case, each individual value was normalized with respect to the global distribution of its corresponding variable across the entire cohort. Specifically, for each continuous feature, we identified the minimum and maximum values in the dataset and applied the following transformation to each athlete’s data:

$$x_{i,norm}=\frac{x_{i}-x_{min}}{x_{max}-x_{min}},$$ (1)

where *x* represents the original value, *x*_*min*_ and *x*_*max*_ are the minimum and maximum values of the feature, and *x*_*i*,*norm*_ is the normalized value. This transformation maps all values to the [0,1] interval, making them directly comparable with one another and mitigating the distortion caused by features with inherently larger ranges.

#### Clustering.

In the clustering phase, we applied agglomerative HC, starting with each athlete as a separate cluster. The algorithm iteratively merges the closest clusters based on a specific distance metric and linkage criterion [32]. This study’s metrics and linkage criteria are detailed in Tables 1 and 2.

**Table 1:** Metrics employed to calculate the distance between two generic elements $p_{a}=(p_{a,1}\;,\;p_{a,2}\;,\ldots \;,\;p_{a,m})$ and $p_{b}=(p_{b,1}\;,\;p_{b,2}\;,\ldots \;,\;p_{b,m})$ [27].

Metric	Formula
Euclidean	$\|p_{a}-p_{b}{\|}_{2}=\sqrt{\sum_{i=1}^{m}(p_{a,i}-p_{b,i}){}^{2}}$
Squared Euclidean	$\|p_{a}-p_{b}{\|}_{2}^{2}=\sum_{i=1}^{m}(p_{a,i}-p_{b,i}){}^{2}$
Cityblock	$\left\|p_{a}-p_{b}{\|}_{1}=\sum_{i=1}^{m}|p_{a,i}-p_{b,i}\right|$
Chebyshev	$\left\|p_{a}-p_{b}{\|}_{\infty }=\max_{i}|p_{a,i}-p_{b,i}\right|$
Correlation	$\|p_{a}-p_{b}{\|}_{\text{corr}}=1-\frac{\sum_{i=1}^{m}(p_{a,i}-{\bar{p}}_{a})(p_{b,i}-{\bar{p}}_{b})}{\sqrt{\sum_{i=1}^{m}(p_{a,i}-{\bar{p}}_{a}){}^{2}}\sqrt{\sum_{i=1}^{m}(p_{b,i}-{\bar{p}}_{b}){}^{2}}}$

**Table 2:** Linkage criteria used to determine the distance between two generic clusters *A* and *B.* Here, |A| and |B| denote the clusters’ cardinality (number of patients), while *C*_*A*_ and *C*_*B*_ represent their centroids [27].

Linkage Criterion	Formula
Single	$\min\{d(p_{a},p_{b}):p_{a}\in A,p_{b}\in B\}$
Complete	$\max\{d(p_{a},p_{b}):p_{a}\in A,p_{b}\in B\}$
Average	$\frac{1}{\left|A||B\right|}\sum_{a\in A}\sum_{b\in B}d(p_{a},p_{b})$
Centroid	$C_{A}-C_{B}$

To illustrate the algorithm’s functionality, consider an example with three clusters (A, B, and C) at the *x*_*th*_ iteration. Cluster A contains two elements, *p*_1_ and *p*_2_, cluster B includes three components, *p*_3_, *p*_4_, and *p*_5_, while cluster C consists of a single element, *p*_6_. During this iteration, one of the cluster combinations (A–B, A–C, or B–C) is selected for merging.

For instance, using the “euclidean” metric and the “single” linkage criterion, the Euclidean distances for the A–B combination are calculated as follows: $d(p_{1},p_{3})$, $d(p_{1},p_{4})$, $d(p_{1},p_{5})$, $d(p_{2},p_{3})$, $d(p_{2},p_{4})$, and $d(p_{2},p_{5})$. With the single-linkage method, the minimum distance among these values is selected. At this stage, each cluster combination is represented by a single entity, and the pair of clusters with the smallest distance is merged. The clustering process was implemented using MATLAB, explicitly leveraging the “linkage” function.

#### Dendrogram analysis and cutting.

**Fig 1:**
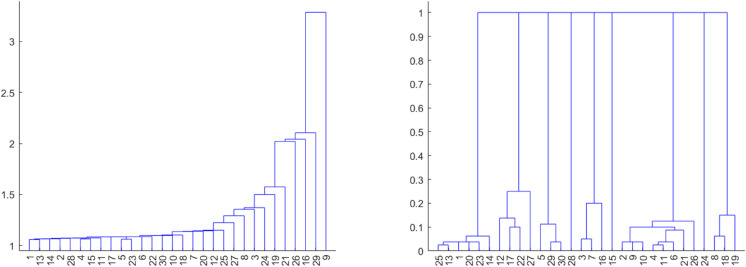
Hierarchical clustering dendrograms with different metrics and datasets. Dendrogram obtained using the variables from **dataset_L_** with Metric “Cityblock” and Linkage “Single” (left image). From **dataset_LE_** with Metric “Euclidean” and Linkage “Single” (right image).

After clustering, the dendrogram was analyzed to identify the optimal number of clusters (Fig 1). The tree-cutting procedure, which involves selecting the point at which to “cut” the tree, was guided by an automatic rule based on the *R*_*k*_ index described right after, which measures the ratio between minimal inter-cluster and maximal intra-cluster variability.

Intra-cluster variability is calculated as the sum of Euclidean distances of each element from its cluster centroid, divided by the cluster size:

$$Var_{intra,A}=\frac{\sum_{j=1}^{\left|A\right|}\sqrt{\sum_{i=1}^{m}(p_{j,i}-C_{A,i}{)}^{2}}}{\left|A\right|},$$ (2)

where *C*_*A*_ is the centroid of cluster *A*. Inter-cluster variability between two clusters is calculated as the Euclidean distance between their centroids:

$$Var_{inter,A,B}=\sqrt{\sum_{i=1}^{m}(C_{A,i}-C_{B,i}{)}^{2}}.$$ (3)

The *R*_*k*_ index is computed as the ratio of the minimal inter-cluster variability to the maximal intra-cluster variability during each iteration:

$$R_{k}=\frac{Var_{inter,MIN}}{Var_{intra,MAX}},$$ (4)

with

$$Var_{intra,MAX}=\max(Var_{intra,A})$$ (5)

$$Var_{inter,MIN}=\min(Var_{intra,A,B}).$$ (6)

**Fig 2:**
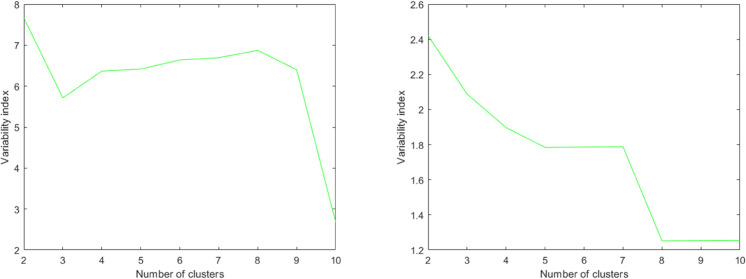
*R*_*k*_ index evolution for different metrics and datasets. *R*_*k*_ index for the last ten iterations using the Cityblock metric and Single linkage from **dataset_L_** (left image), and the Euclidean metric and Single linkage from **dataset_LE_** (right image). In both cases, the best *R*_*k*_ index occurs with two clusters.

The tree was cut at the point where the *R*_*k*_ value was maximized, indicating the most meaningful clustering configuration (Fig 2).

The resulting clusters were interpreted as distinct SCD risk states, each representing a prototype of patients with similar health characteristics.

#### Clustering patterns analysis.

Clustering aims to achieve an unsupervised classification based on features related to the subject of study. The identified clusters may have physical significance, representing meaningful groupings within the data. In particular, the smaller clusters may suggest a higher correlation with the risk of experiencing sudden cardiac death (SCD). The clustering analysis conducted on **dataset_L_** (8 features) and **dataset_LE_** (45 features) revealed that various metrics and similarity criteria often produced identical or highly similar clusterings.

Specifically, for **dataset_L_**, only four distinct clustering patterns were identified among 37 patients, with the minority clusters frequently involving the same type of individuals. For **dataset_LE_**, 10 different clustering patterns emerged; however, two were deemed insignificant in the context of sudden death analysis. These two clusterings produced configurations with one minority cluster of 332 patients (using the complete linkage method with Euclidean, squared Euclidean, and Minkowski metrics) and another with 78 patients (using complete linkage and the Minkowski metric). The complete Chebyshev clustering result was also excluded due to the *R*_*k*_-factor diverging with increasing clusters. Consequently, seven valid clusterings were considered for **dataset_LE_**, involving 33 patients. The clustering patterns are evident in Tables 3 and 4, which report the number of clusters obtained for various metrics and similarity criteria.

**Table 3:** Number of clusters obtained using different metrics and similarity criteria: (dataset_L_). Cells with the same color indicate coincident clusterings.

	Euclidean	Squared Eucl.	Cityblock	Chebychev	Minkowski	Spearman
Single	2	2	2	8	2	2
Complete	9	9	9	2	9	2
Average	9	9	9	8	9	2
Centroid	9	9	9	8	9	2

**Table 4:** Clustering results for different metrics and similarity criteria: (dataset_LE_). Matching clusterings are highlighted with the same color. Excluded clusterings are represented by numbers followed by a comma (where the second number indicates the smallest cluster size) or marked as “div” to denote divergence of the *R*_*k*_ coefficient.

	Euclidean	Squared Eucl.	Cityblock	Chebychev	Minkowski	Spearman
Single	2	2	2	6	2	5
Complete	2,332	2,332	2	Div	2,332	2,78
Average	2	2	2	3	2	2
Centroid	2	2	2	3	2	2

**Fig 3:**
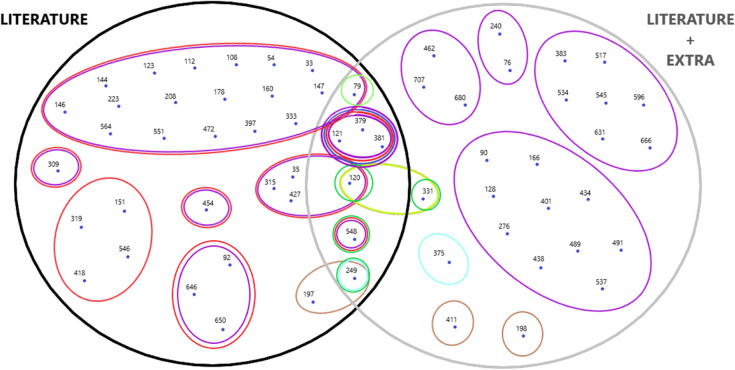
Euler-Venn diagrams representing the identified patients’ clusterings of interest. These diagrams offer a graphical visualization of the relationships between clusters, complementing the tabular data in Table 5.

Cells with the same color indicate coincident clusterings, while cells with numbers followed by a comma and another number or cells marked with “div” indicate excluded clusterings. The number to the right of the comma represents the size of the smallest cluster, while “div” indicates the divergence of the *R*_*k*_ coefficient. Seven patients appeared in common clusters across the clusterings derived from both datasets. Therefore, out of 711 analyzed patients, sixty-four were identified as “different” compared to the rest. These patients, distributed across eleven total clusterings, were analyzed further. Table 5 presents the results of the clusterings based on the parameters. In addition, Table 5 and Fig 3 offer a tabular and graphical representation of the clusterings of interest for the patients using Euler-Venn diagrams. Notably, these sixty-four individuals exhibited distinct characteristics.

**Table 5:** Tabular representation of the clusterings of interest for the identified patients. Among the 711 analyzed patients, 64 were classified as “different” and distributed across 11 clusterings. This table provides a detailed overview of their distribution. The abbreviations “e”, “se”, “ci”, “ch”, “mi”, and “sp” stand for Euclidean, Squared Euclidean, Cityblock, Chebyshev, Minkowski, and Spearman, respectively.

Clusterings		(*cluster*_*i*_) Patient IDs
**Literature**		
1	Single (e, se, ci, mi), Complete (ch)	(1) 121, 379, 381
2	Single (ch), Average (ch), Centroid (ch)	(1) 92, 646, 650, (2) 548, (3) 121, 379, 381, (4) 309, (5) 33, 54, 79, 108, 112, 123, 144, 146, 147, 160, 178, 208, 223, 333, 397, 472, 551, 564, (6) 454, (7) 35, 120, 315, 427
3	Single (sp), Complete (sp), Average (sp), Centroid (sp)	(1) 197, 249
4	Complete, Average, Centroid (e, se, ci, mi)	(1) 92, 646, 650, (2) 548, (3) 121, 379, 381, (4) 309, (5) 33, 54, 79, 108, 112, 123, 144, 146, 147, 160, 178, 208, 223, 333, 397, 472, 551, 564, (6) 454, (7) 35, 120, 315, 427, (8) 151, 319, 418, 546
**Literature + Extra**		
5	Single (e, se, ci, mi), Average (e, se, ci, mi), Centroid (e, se, ci, mi)	(1) 120
6	Single (ch)	(1) 462, 680, 707, (2) 76, 240, (3) 121, 379, 381, (4) 383, 517, 534, 545, 596, 631, 666, (5) 90, 128, 166, 276, 401, 434, 438, 489, 491, 537
7	Complete (ci)	(1) 120, 331
8	Average (ch)	(1) 411, (2) 198
9	Average (sp), Centroid (sp)	(1) 79
10	Centroid (ch)	(1) 375, (2) 249
11	Single (sp)	(1) 249, (2) 548, (3) 120, (4) 331

They are classified as Class C athletes with a familial and/or personal history of cardiopathy. Typical findings among them include atrioventricular block on both resting and stress ECG, QT segment prolongation during stress ECG, right axis deviation on resting and stress ECG, tachycardia on both resting and stress ECG, and ventricular pre-excitation seen on either or both types of ECG. Additional traits may include being born prematurely via cesarean section, complete right bundle branch block observed on stress ECG, hypertension with T-wave inversion in inferior and lateral leads on stress ECG, or a combination of these conditions. While the clustering results provided intriguing insights, several challenges emerged. First, the data was analyzed across eight or forty-five variables, predominantly binary (*Y/N*), making interpretation difficult. Second, despite their interpretative interest, the results were challenging to present practically and graphically. To address this, clustering results were visualized using PCA for both datasets. PCA was also applied to a subset of selected features, and a detailed discussion is provided in the section dedicated to PCA analysis.

## Principal component analysis for graphical representation

Principal Component Analysis (PCA) is a dimensionality reduction technique that simplifies complex datasets by transforming the original data into a new set of variables called principal components. These components are linear combinations of the original variables and are ordered such that the first principal component captures the most significant variance, followed by the second, which captures the next most considerable variance, and so on. Mathematically, PCA involves the following steps. First, the data is standardized by subtracting the mean and dividing by the standard deviation for each variable:

$$z=\frac{x-\mu }{\sigma }.$$ (7)

This ensures that all variables contribute equally to the analysis. Next, the covariance matrix Σ is computed to quantify the relationships between variables:

$$\Sigma =[\begin{array}{ccc} Cov(x,x) & Cov(x,y) & Cov(x,z)\\ Cov(y,x) & Cov(y,y) & Cov(y,z)\\ Cov(z,x) & Cov(z,y) & Cov(z,z) \end{array}]$$ (8)

where:

$$Cov(x,y)=\frac{1}{n}\sum_{i=1}^{n}(x_{i}-\mu_{x})(y_{i}-\mu_{y}).$$ (9)

For the covariance matrix, the eigenvectors $\nu_{i}$ indicate the directions of the data’s maximum variance (the principal components), while the eigenvalues $\lambda_{i}$ represent the magnitude of variance along each eigenvector. The principal components are ranked by their eigenvalues in descending order. A feature vector is created by selecting the eigenvectors corresponding to the largest eigenvalues, which retain the components while reducing dimensionality and preserving most of the information. The final step involves reorienting the data along the principal component axes using the transformation (see [33]):

$$Final\;Dataset=Feature\;Vector^{T}*Standardized\;Original\;Dataset^{T}$$ (10)

In clustering analysis, PCA is beneficial for visualizing high-dimensional data. Applying PCA to a dataset containing different clusters of patients, each described by multiple features, can reduce the feature space to two or three dimensions, making it easier to visualize. When plotting the principal components, each data point can be assigned a color corresponding to its cluster membership. This allows researchers to observe whether the points in the same cluster are positioned close to each other and far from points in other clusters. The clustering algorithm successfully captures the data’s inherent structure if clusters appear well-separated in the principal component space. Moreover, when clusters appear differentiated in the PCA representation, this approach can also be extended to include new patients. By projecting the data of new individuals onto the same PCA-transformed space, their positions relative to existing clusters can be visualized. This provides a clear, intuitive way to assess how a new patient aligns with specific clusters, which may have physical or clinical significance. This ability highlights PCA, particularly the feature vectors, as a powerful data representation tool, facilitating dimensionality reduction and the interpretation of new data within existing patterns and structures. This study observed a significant overlap between many clusters in the 11 identified clusterings. As a result, the cluster information was consolidated for representation. For both datasets, **dataset_L_** and **dataset_LE_**, the common clusters were merged, leading to a total of 21 clusters: 10 derived from the clustering of **dataset_L_** and 11 from the clustering of **dataset_LE_**. The information on the clusters and the respective patients identified through the consolidation process is presented in Table 6. The patient IDs in bold correspond to those included in clusters identified from both **dataset_L_** and **dataset_LE_**.

**Table 6:** Consolidated clustering results from dataset_L_ and dataset_LE_, resulting in 21 clusters. Bold patient IDs represent those in clusters identified from both datasets. The term “others” indicates the rest of the patients in the dataset.

Clusterings	(*cluster*_*i*_) Patients IDs
**Literature**	
Final Clustering Proposal	(1) Others, (2) 33, 54, **79**, 108, 112, 123, 144, 146, 147, 160, 178, 208, 223, 333, 397, 472, 551, 564, (3) **121, 379, 381**, (4) 454, (5) 197, **249**, (6) 92, 646, 650, (7) 35, **120**, 315, 427, (8) 309, (9) 151, 319, 418, 546, (10) **548**
**Literature + Extra**	
Final Clustering Proposal	(1) 383, 517, 534, 545, 596, 631, 666, (2) **120**, (3) **249**, 331, 375, **548**, (4) 198, (5) 411, (6) **79**, (7) 76, 240, (8) Others, (9) **121, 379, 381**, (10) 462, 680, 707, (11) 90, 128, 166, 276, 401, 434, 438, 489, 491, 537

PCA was then applied to the two datasets, and the final clusters (i.e., the overall clusters) were visualized by assigning different colors to the data points of each cluster on the principal component plots. The data was projected onto the first two principal components for visualization in both cases. The results showed that, in most cases, the elements of the clusters are positioned in distinct regions of the plot, clearly separating the clusters.

**Fig 4:**
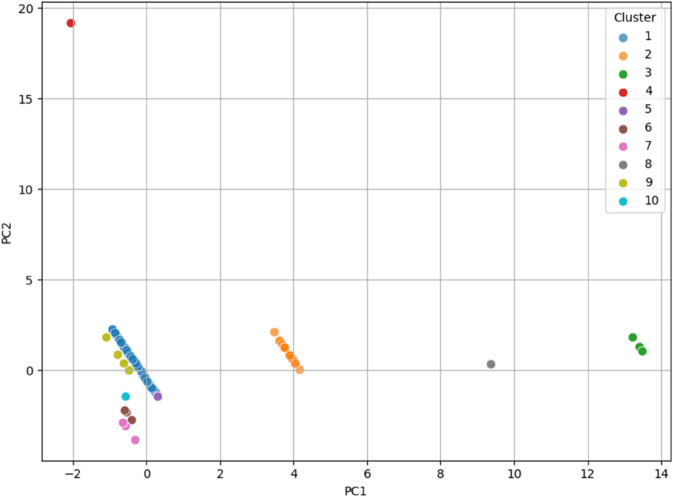
PCA visualization of the final clusters for dataset_L_, with data points colored according to their respective clusters. The first two principal components separate the clusters, with data points in **dataset_L_** organizing along parallel, linearly separable regions, suggesting potential for linear classification in the PCA-transformed space.

Interestingly, in the case of **dataset_L_**, the data points of the patients appeared to organize themselves along parallel regions, which were linearly separable. This suggests that the clusters in **dataset_L_** are well-structured and can potentially be discriminated with a linear classifier in the PCA-transformed space. The only exception is represented by Cluster 5, which is composed of just two elements that fall within the lower part of the band formed by the elements of Cluster 1, thus partially overlapping with it. The plot for the first two principal components of **dataset_L_** is shown in Fig 4.

**Fig 5:**
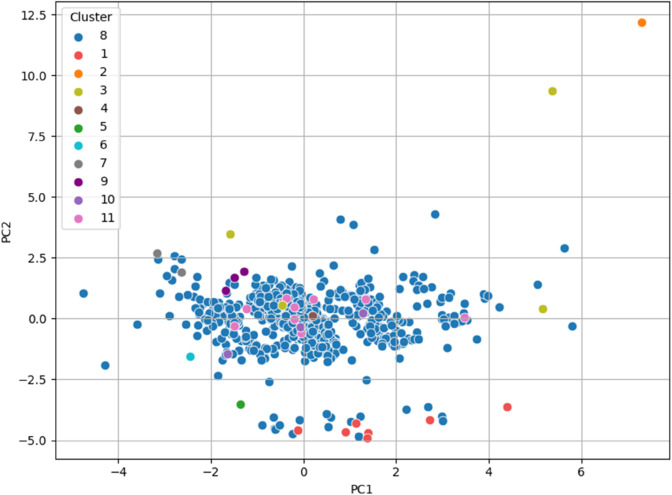
PCA visualization of the final clusters for dataset_LE_, with data points colored according to their respective clusters. The elements of the clusters are arranged peripherally around the central data points, indicating a different underlying structure, where clusters form at the edges of the dataset.

On the other hand, for **dataset_LE_**, the elements belonging to the clusters tended to arrange themselves peripherally relative to the rest of the sample. This indicates a different underlying structure, where clusters form around the edges of the dataset, separating themselves from the central data points. An exception to this pattern is observed for all the elements of clusters 4, 10, and 11, as well as one element from Cluster 3, which are instead located closer to the central region of the plot. The plot for the first two principal components of **dataset_LE_** is shown in Fig 5. This differentiation in the clusters’ spatial organization highlights the effectiveness of PCA in revealing structural patterns in the data.

By visualizing the clustering results in the principal component space, it becomes possible to better understand the relationships between the clusters and the data distribution and even to hypothesize about the physical or clinical significance of these patterns. The eigenvalues and eigenvectors are presented in Table 15.

## Discussion and application

### Interpretation of findings

This study introduces a novel data-driven methodology for assessing SCD risk in athletes by integrating unsupervised HC with PCA. Traditional screening methods rely on clinical guidelines and predefined risk factors. In contrast, this approach identifies natural groupings in high-dimensional data, offering a more nuanced stratification of athletes based on shared physiological and clinical characteristics. By leveraging clustering, the study uncovers hidden patterns in athlete profiles, distinguishing subgroups that may correspond to varying levels of SCD risk. Principal component analysis further enhances interpretability by reducing dimensionality, allowing for a visual representation of risk groupings in a lower-dimensional space. This combination offers an innovative perspective on SCD risk assessment, complementing standard pre-participation screenings [34].

The clustering results align with previous research on SCD risk stratification, supporting the notion that a small subset of individuals consistently emerges as distinct, potentially corresponding to an elevated cardiovascular risk. These clusters were medically interpretable as individuals in high-risk groups exhibited characteristics associated with known clinical predictors of SCD, such as electrocardiographic abnormalities, hypertrophic markers, and family history of cardiovascular disease [35]. The analysis of the two datasets showed clear differences in high-risk groups of athletes.

In the **dataset_L_**, we observed more frequent ECG abnormalities. Specifically, 10.8% of these athletes had T-wave inversions in the lateral or inferolateral leads of the resting ECG, and 2.7% showed this in the anterior leads. Additionally, 10.8% had prolonged QTc intervals on the stress ECG, and a significant 56.8% showed signs of ventricular pre-excitation. Notably, syncope (fainting) occurred in 8.1% of high-risk individuals, while none in the low-risk group reported this. The high-risk group also exhibited slightly lower average body mass index (BMI), at 20.86 compared to 22.04, and a lower average heart rate (72.05 bpm vs. 75.05 bpm), indicating different physiological patterns.

In the **dataset_LE_**, similar trends were noted. Here, 57.6% of individuals in the high-risk group had a family history of heart disease, in contrast to 19.3% in the low-risk group. A personal history of heart disease was seen in 51.5% of high-risk individuals, compared to just 4.4% in the low-risk group. Furthermore, the high-risk subgroup exhibited higher rates of sinus tachycardia (24.2% vs. 2.8%) and ventricular pre-excitation (12.1% vs. 2.5%) on the resting ECG. Interestingly, the average heart rate was significantly higher in the high-risk group (80.2 bpm vs. 74.6 bpm). Some features, like pectus excavatum and certain axis deviations, were actually more common in the low-risk group, which could suggest some protective factors or random variability.

These differences are summarized in Tables 7 and 8, which present only those features showing statistically significant differences between the high-risk and low-risk groups for each dataset. **dataset_L_** included 37 high-risk and 674 low-risk individuals, while **dataset_LE_** comprised 33 high-risk and 678 low-risk individuals, respectively.

**Table 7:** Comparison of feature prevalence in risk and no-risk clusters for dataset_L_ and dataset_LE_. Variables from the original literature-based set ( **dataset_L_**) are shown in bold in the leftmost column. Values with statistically significant differences within each dataset are also indicated in bold. Feature numbers correspond to those reported in Table 14.

Feature	Literature		Literature + Extra	
	Risk	No Risk	Risk	No Risk
(2) Family History of Heart Disease	16.21%	21.36%	**57.57%**	19.32%
(3) Pesonal History of Heart Disease	**10.81%**	6.37%	**51.51%**	4.42%
**(4) Syncope**	**8,10%**	**0%**	**0%**	**0.44%**
(6) CAESAREAN SECTION: Premature Delivery	0%	0.74%	**6.02%**	0.44%
(7) Pectus Excavatum	0%	**0.44%**	0%	**0.44%**
(10) Blood Pressure	**2.70%**	0.89%	**3.03%**	0.89%
(12) Sinus Tachycardia (Resting ECG)	2.71%	3.85%	**24.24%**	2.80%
(14) Atrial Arrhythmia (Resting ECG)	0%	**1.18%**	0%	**1.17%**
(17) Complete Right Bundle Branch Block (Resting ECG)	**2.70%**	0%	**3.03%**	0%
(18) Atrioventricular Block (Resting ECG)	**2.70%**	0.44%	**6.06%**	0.29%
(19) Right Axis Deviation (Resting ECG)	0%	1.78%	**6.06%**	1.46%
**(21) T-wave Inversion in the Lateral or Inferolateral Leads (Resting ECG)**	**10.81%**	**0%**	**0.58%**	**1.17%**
**(22) T-wave Inversion in the Anterior Leads (Resting ECG)**	**2.70%**	**0%**	**0%**	**1.14%**
**(24) Ventricular pre-excitation (Resting ECG)**	**10.81%**	**0%**	**9.09%**	**0.14%**
(34) Complete Right Bundle Branch Block (Stress ECG)	0%	0.59%	**3.03%**	0.44%
(36) Atrioventricular Block (Stress ECG)	**2.70%**	0.29%	**6.06%**	0.14%
(37) Right Axis Deviation (Stress ECG)	0%	2.37%	**60.60%**	2.06%
**(38) T-wave Inversion in the Lateral or Inferolateral Leads (Stress ECG)**	**2.70%**	**0%**	**3.03%**	**0%**
(39) T-wave Inversion in the Anterior Leads (Stress ECG)	**5.49%**	0.44%	0%	0.73%
(41) Ventricular pre-excitation (Stress ECG)	**56.75%**	0%	**12.12%**	2.50%
(43) Prolonged QTc (Stress ECG)	**10.81%**	0%	**3.03%**	0.44%

**Table 8:** Comparison of feature statistics in risk and no-risk clusters for dataset_L_ and dataset_LE_. Feature numbers correspond to those reported in Table 14.

Feature	Average		Standard Deviation	
	Risk	No Risk	Risk	No risk
(8) Body Mass Index (numeric)				
Literature	20.86%	**22.04%**	5.46%	4.07%
Literature + Extra	21.62%	21.99%	4.50%	4.14%
(11) Heart Rate				
Literature	72.05%	75.05%	13.54%	13.87%
Literature + Extra	**80.24%**	74.63%	20.57%	13.42%

Analyzing the distinct compositions of individual clusters within the high-risk group offers significant clinical insights, enhancing our understanding of the risk class’s internal structure. This nuanced analysis enables healthcare professionals to interpret how specific feature combinations relate to cluster placements in PCA space, thereby refining diagnostic strategies. Tables 9 and 10 illustrate representative configurations of features found in high-risk clusters within **dataset_L_**, while Tables 11 and 12 refer to **dataset_LE_**.

**Table 9:** Feature distribution across clusters for dataset_L_. Columns indicate feature numbers as listed in Table 14 or Table 10. Rows represent groups of cluster elements that share the same feature activation pattern, as listed in Table 6. Numbers in parentheses denote the number of individuals within each subgroup (or pattern). When these numbers are shown in bold, they identify elements also found in **dataset_LE_**; when they are underlined, they indicate elements located in the PCA region defined as non-risk.

Cluster	Feature																
	1	2	3	4	10	12	16	18	21	22	24	30	36	38	39	41	43
**Cluster 2**																	
144	✓											✓		✓	✓		
472	✓					✓						✓				✓	
33, 123..(4)	✓											✓				✓	
54, 551	✓															✓	
146	✓		✓								✓	✓					
**79**	** ✓ **											** ✓ **				** ✓ **	
333		✓										✓				✓	
397, 564		✓														✓	
108, 112, 208	✓															✓	
178	✓	✓														✓	
223	✓	✓										✓				✓	
**Cluster 3**																	
**121,379,381**	** ✓ **										** ✓ **					** ✓ **	
**Cluster 4**																	
454										✓							
**Cluster 5**																	
197	✓																
249							✓										
**Cluster 6**																	
92,646,650	✓			✓													
**Cluster 7**																	
35	✓											✓					✓
**120**			** ✓ **					** ✓ **					** ✓ **				** ✓ **
315, 427												✓					✓
**Cluster 8**																	
309	✓		✓								✓						
**Cluster 9**																	
151,319,418	✓								✓								
546	✓	✓							✓			✓					
**Cluster 10**																	
548	✓				✓							✓		✓			

**Table 10:** Representative feature patterns across high-risk clusters for dataset_L_. Rows correspond to features, as listed in Table 14. Columns represent cluster IDs, as defined in Table 6, and indicate all features activated by at least one element within each cluster.

Feature	Cluster								
	2	3	4	5	6	7	8	9	10
(1) Class C Sport	✓	✓		✓	✓	✓	✓	✓	✓
(2) Family History of Heart Disease	✓							✓	
(3) Pesonal History of Heart Disease	✓					✓	✓		
(4) Syncope					✓				
(10) IPER Blood Preassure									✓
(12) Sinus Tachycardia (Resting ECG)	✓								
(16) Complete Right Bundle Branch Block (Resting ECG)				✓					
(18) Atrioventricular Block (Resting ECG)						✓			
(21) T-wave Inversion in the Lateral or Inferolateral Leads (Resting ECG)								✓	
(22) T-wave Inversion in the Anterior Leads (Resting ECG)			✓						
(24) Ventricular pre-excitation (Resting ECG)	✓	✓					✓		
(30) Sinus Tachycardia (Stress ECG)	✓					✓		✓	✓
(36) Atrioventricular Block (Stress ECG)						✓			
(38) T-wave Inversion in the Lateral or Inferolateral Leads (Stress ECG)	✓								
(39) T-wave Inversion in the Anterior Leads (Stress ECG)	✓								✓
(41) Ventricular pre-excitation (Stress ECG)	✓	✓							
(43) Prolonged QTc (Stress ECG)						✓			

**Table 11:** Feature distribution across clusters for dataset_LE_. Columns indicate feature numbers as listed in Table 14 or Table 12. Rows represent groups of cluster elements that share the same feature activation pattern, as listed in Table 6. Numbers in parentheses denote the number of individuals within each subgroup (or pattern). When these numbers are shown in bold, they identify elements also found in **dataset_L_**; when they are underlined, they indicate elements located in the PCA region defined as non-risk.

Cluster	Feature																
	1	2	3	6	10	12	16	18	19	24	30	34	36	37	38	41	43
**Cluster 1**																	
517,534,..(6)	✓	✓				✓					✓						
383	✓	✓				✓											
**Cluster 2**																	
**548**	** ✓ **				** ✓ **						** ✓ **				** ✓ **		
**120**			** ✓ **					** ✓ **					** ✓ **				** ✓ **
**Cluster 3**																	
331	✓	✓						✓					✓				
375	✓			✓			✓										
249							✓										
548	✓				✓						✓				✓		
**Cluster 4**																	
198	✓	✓	✓	✓													
**Cluster 5**																	
411			✓			✓											
**Cluster 6**																	
**79**			** ✓ **								** ✓ **					** ✓ **	
**Cluster 7**																	
76,240									✓					✓			
**Cluster 9**																	
**121,379,381**	** ✓ **									** ✓ **						** ✓ **	
**Cluster 10**																	
462,680,707			✓								✓						
**Cluster 11**																	
90,128,..(10)	✓	✓	✓														

**Table 12:** Representative feature patterns across high-risk clusters for dataset_LE_. Rows correspond to features, as listed in Table 14. Columns represent cluster IDs, as defined in Table 6, and indicate all features activated by at least one element within each cluster.

Feature	Cluster									
	**1**	**2**	**3**	**4**	**5**	**6**	**7**	**9**	**10**	**11**
(1) Class C Sport	✓	✓	✓	✓				✓		✓
(2) Family History of Heart Disease	✓		✓	✓						✓
(3) Pesonal History of Heart Disease		✓		✓	✓	✓			✓	✓
(6) CAESAREAN SECTION: Premature Delivery			✓	✓						
(10) IPER Blood Preassure		✓								
(12) Sinus Tachycardia (Resting ECG)	✓				✓					
(16) Complete Right Bundle Branch Block (Resting ECG)			✓							
(18) Atrioventricular Block (Resting ECG)		✓	✓							
(19) Right Axis Deviation (Resting ECG)							✓			
(24) Ventricular pre-excitation (Resting ECG)								✓		
(30) Sinus Tachycardia (Stress ECG)	✓	✓				✓			✓	
(34) Complete Right Bundle Branch Block (Stress ECG)										
(36) Atrioventricular Block (Stress ECG))		✓	✓							
(37) Right Axis Deviation (Stress ECG)							✓			
(38) T-wave Inversion in the Lateral or Inferolateral Leads (Stress ECG)		✓								
(41) Ventricular pre-excitation (Stress ECG)						✓		✓		
(43) Prolonged QTc (Stress ECG)		✓								

For instance, in **dataset_L_**, Cluster 2 is notable for its inclusion of athletes presenting with Class C sport participation, conditions such as sinus tachycardia and ventricular pre-excitation (Stress ECG), and prevalent T-wave inversions in anterior or lateral leads. Coupled with personal and family histories of heart disease, this cluster highlights critical profiles requiring targeted clinical intervention. Conversely, Cluster 7 reveals rare but alarming profiles characterized by prolonged QTc and syncope, indicating an urgent need for thorough evaluation and monitoring. A closer look at Cluster 5 suggests a different clinical narrative, as its members exhibit minimal activations across descriptive variables. This potentially identifies them as a cohort of low-risk subjects. In the PCA projection, these individuals are situated at the lower boundary of Cluster 1, which similarly includes low-risk cases. Merging these clusters could enhance the interpretation of subjects exhibiting a non-risk profile.

Turning to **dataset_LE_**, Cluster 1 distinctly showcases a pattern of Class C sport participation, family history of heart disease, and sinus tachycardia readings from Resting ECG. These interrelated patterns underscore the importance of considering both individual features and specific combinations when determining risk groupings in PCA space. Similarly, Clusters 10 and 11 present low levels of feature activation, indicating a prevalence of non-risk profiles. Their positioning within the central region of Cluster 8 suggests potential for consolidation, reinforcing the clinical approach of distinguishing between at-risk and non-risk groups. However, Clusters 3 and 4 present unique challenges. Cluster 3, in particular, presents interpretive challenges for two main reasons: (i) its members are spatially distributed across different regions in the PCA plot, indicating internal heterogeneity; and (ii) element 375, despite showing a moderate number of activations, is positioned centrally within the band formed by Cluster 8—suggesting potential non-risk status. A similar ambiguity arises with element 198 from Cluster 4, which also falls in the dense central region dominated by Cluster 8 elements despite exhibiting numerous feature activations. Since this area also includes several members of Cluster 11, it may be interpreted as either at-risk or non-risk, depending on the severity and clinical relevance attributed to the overlapping features in relation to SCD.

All analytical tools employed to produce the visual results shown in Figs 4 and 5 are accessible in the Experiment subfolder of the central repository at https://github.com/hyacintus/Sudden-Cardiac-Death-Survey/tree/26ba71a0a69e5de58d015f1368512e99ba99dc03/Experiment. This comprehensive analysis underscores the importance of cluster evaluation in driving informed clinical decisions and advancing patient care.

However, this study diverges from conventional models in several key ways. Unlike traditional methods that assess risk factors individually, this model analyzes their combined interactions, offering a more comprehensive risk assessment. Most prior studies rely on supervised classification models trained on known SCD cases; here, clustering provides an unbiased grouping, potentially identifying previously unrecognized risk profiles. While prior research establishes SCD risk factors, the graphical PCA representation is unique, offering a dynamic decision-support tool for sports cardiologists [36].

### Testing the model on new data

There are several potential approaches to building a classifier based on the data used for clustering and subsequent PCA in this study. Among the viable methods are fuzzy logic systems [37], artificial neural networks [38], k-means-based classification strategies [39], or even the direct use of the similarity metrics and distance measures already described in this work [32], combined and adapted for classification purposes. However, initiating the development of such a classifier at this stage would be premature and of limited utility, given that the internal characteristics of the identified clusters have not yet been longitudinally studied or clinically validated over time through dedicated follow-up investigations.

**Fig 6:**
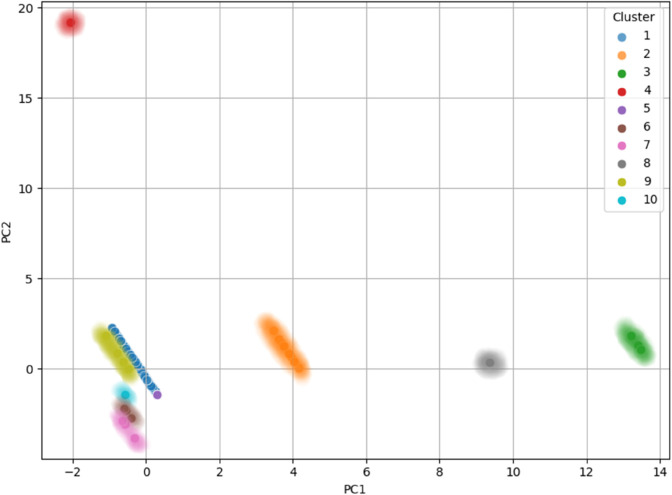
PCA visualization of the final clusters for dataset_L_, with data points colored according to their respective clusters. Additionally, the corresponding hypothetical cluster membership regions are shown using the same colors, highlighting the areas each cluster occupies in the PCA space.

**Fig 7:**
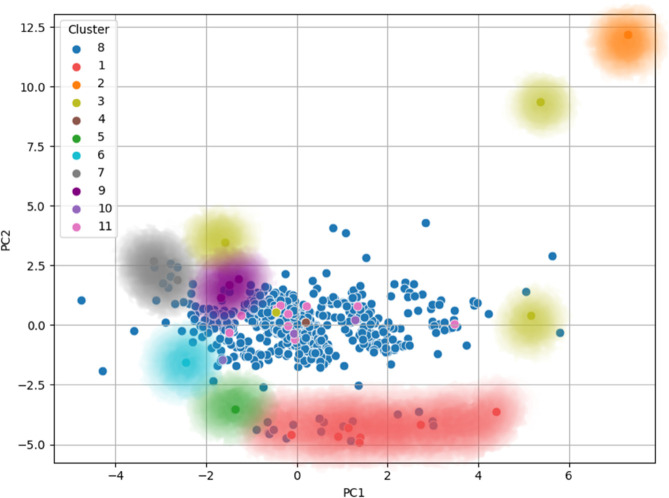
PCA visualization of the final clusters for dataset_LE_, with data points colored according to their respective clusters. Additionally, the corresponding hypothetical cluster membership regions are displayed using the same colors, emphasizing the peripheral arrangement of clusters around the central data points. This suggests a distinct underlying structure, where clusters emerge at the edges of the dataset.

Instead, this section aims to demonstrate how new data points—i.e., newly acquired subjects—naturally align with specific clusters in the PCA-transformed space, based on their feature profiles. More precisely, the idea is to show that new elements tend to distribute across the PCA plots in regions where clusters with similar feature combinations are located, as identified in Figs 6 and 7.

To this end, a set of 68 new subjects was introduced. For simplicity and for illustrative purposes, these new subjects were manually labeled as either “at risk” or “not at risk” based on the characteristic features associated with the risk profiles defined in both **dataset_L_** and **dataset_LE_**. According to these criteria, 7 subjects were classified as “at risk” based on the feature structure of **dataset_L_**, and 8 based on that of **dataset_LE_**, with 3 subjects overlapping across both designations.

**Fig 8:**
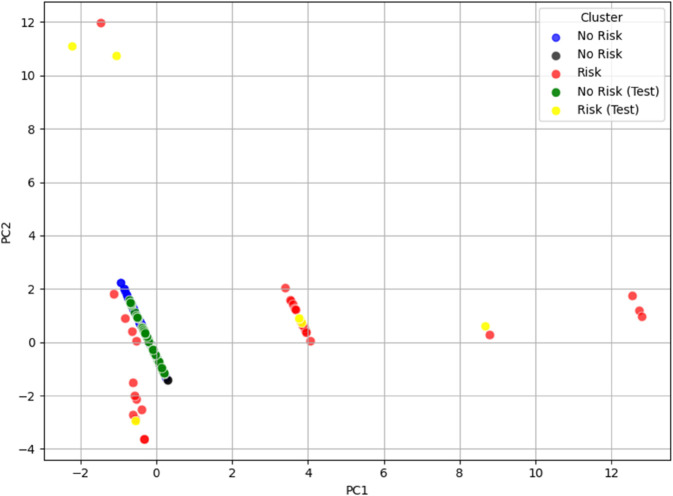
PCA test visualization of the Risk/No Risk clusters for dataset_L_, with data points colored according to their respective clusters. The new subjects are colored yellow (at risk) and green (not at risk), while original points remain red (at risk), blue (not at risk), and black for ambiguous cases (e.g., Cluster 5).

**Fig 9:**
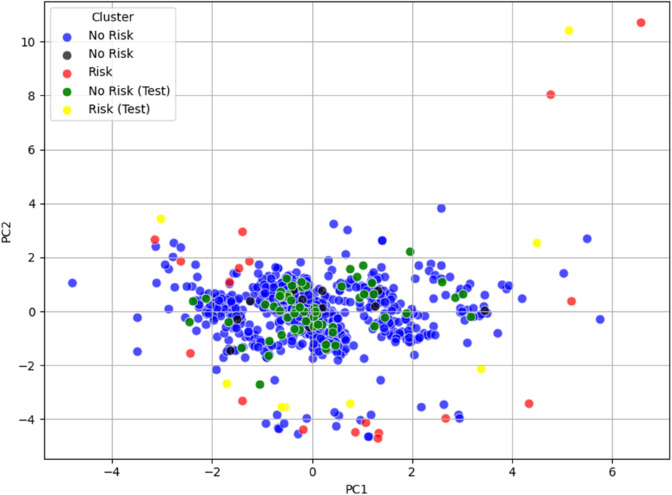
PCA test visualization of the Risk/No Risk clusters for dataset_LE_, with data points colored according to their respective clusters. The new subjects are colored yellow (at risk) and green (not at risk), while original points remain red (at risk), blue (not at risk), and black for ambiguous cases (e.g., Cluster 4, 10 and 11).

The new subjects were then projected into the PCA space of each dataset and visualized alongside the original clustered data. In these plots, shown in Figs 8 and 9, the new subjects are colored in yellow (at risk) and green (not at risk), while the original dataset elements retain their previous labeling: red for at-risk and blue for not-at-risk individuals. As previously discussed, uncertain clusters or ambiguous elements (e.g., Cluster 5 in **dataset_L_**, and Clusters 4, 10, and 11 in **dataset_LE_**) were treated as “not at risk” and are represented in black.

The resulting visualization clearly shows that the new data points tend to occupy the same regions as the original clusters with similar characteristics. This provides preliminary qualitative validation of the cluster configurations, supporting the hypothesis that the PCA space preserves meaningful structural relationships between subjects.

All tools used to generate the visual results presented in Figs 8 and 9 are available in the Experiment subfolder within the main repository at https://github.com/hyacintus/Sudden-Cardiac-Death-Survey/tree/809e6aac122aba71379db23d426af654bda3a944/Test

### Clinical applications and prospects for implementation

This methodology presents a potential real-world application for pre-participation cardiovascular screenings in athletes. Defining an athlete’s profile using either a reduced feature set of eight key variables or the entire dataset of forty-five features, assigning the athlete to a cluster based on distance metrics, and using PCA-based visualization to determine their proximity to high-risk groups offer an intuitive interpretation of their risk status. This approach could augment traditional screening methods, helping sports cardiologists prioritize further testing in athletes who appear closer to high-risk clusters. It could also serve as an early flagging system, directing individuals toward more in-depth cardiac evaluations [40].

Despite its potential, this approach has three key limitations. While high-risk clusters correspond to known clinical markers, there is no definitive proof that they predict actual SCD events. Future studies should conduct external validation using independent datasets incorporating diverse athletic and non-athletic populations to assess model generalizability [41]. Clustering groups individuals with shared characteristics, but does not produce explicit risk equations. Future research should integrate supervised learning models like logistic regression, deep learning, and support vector machines to refine interpretability and predictive power [42]. Principal component analysis reduces dimensionality but lacks direct physiological meaning, meaning proximity to a cluster suggests relative risk rather than absolute thresholds. Incorporating biomarkers such as genetic predisposition indicators and electrocardiographic parameters could enhance clinical applicability [43].

Future research should prioritize prospective cohort studies to enhance and validate this approach, assessing whether clustering-based stratifications correspond with actual SCD incidence. Additionally, optimizing feature selection through advanced techniques, such as recursive feature elimination and Shapley additive explanations, will help isolate the most predictive variables. Incorporating physiological markers, such as cardiac MRI data, genetic risk scores, and exercise-induced ECG responses, can further enhance clinical relevance. Collaborating with experts in sports medicine and cardiology will help refine risk thresholds and enhance real-world applications for both elite and amateur athletes. The integration of clustering and PCA in athlete screening represents a significant shift from traditional, static risk assessment models to a more flexible, data-driven approach. While further validation is required, this methodology offers a promising tool to enhance pre-participation screenings, identify previously undetected risk groups, and ultimately contribute to improved athlete safety. By refining this approach through machine learning advancements, external validation, and clinical collaboration, it has the potential to become an essential component of modern sports cardiology [44–46].

**Fig 10:**
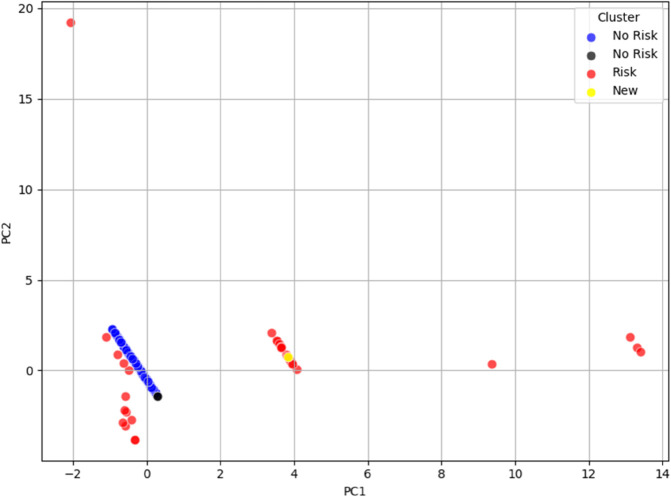
PCA trial visualization of the Risk/No Risk clusters for dataset_L_, with data points colored according to their respective clusters. Original subjects are shown in red (at risk), blue (not at risk), and black for ambiguous cases (e.g., Cluster 5). Newly tested subjects are displayed in yellow.

**Fig 11:**
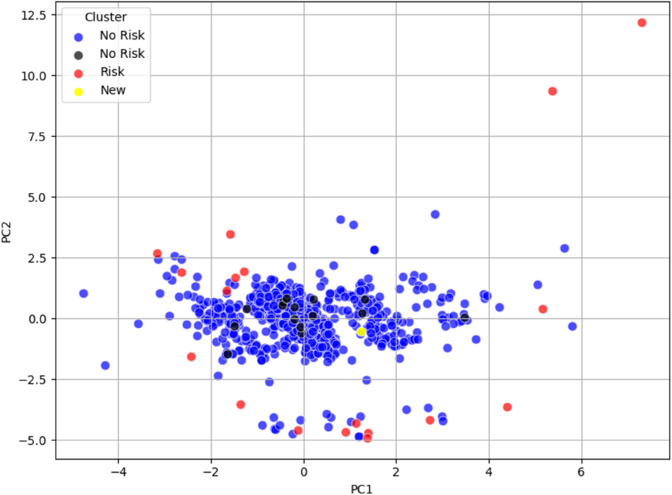
PCA trial visualization of the Risk/No Risk clusters for dataset_LE_, with data points colored according to their respective clusters. Original subjects are shown in red (at risk), blue (not at risk), and black for ambiguous cases (e.g., Clusters 4, 10, and 11). Newly tested subjects are displayed in yellow.

To support wider exploration and independent testing of this approach, a dedicated testing tool is provided in the Experiment subfolder of the main repository at https://github.com/hyacintus/Sudden-Cardiac-Death-Survey/tree/3fc0849814be127676081ef15c8a1903d06d3c9c/Trial. This tool is designed to reproduce visualizations such as those shown in Figs 10 and 11, and includes step-by-step instructions.

To provide clinicians with a cutting-edge advantage in sports cardiology, the integration of advanced data analytics and machine learning approaches can revolutionize the understanding of cardiac health in athletes. By leveraging sophisticated algorithms, clinicians can analyze large datasets effectively, identifying underlying patterns and risk factors that may not be apparent through traditional methods.

- Real-Time Monitoring: Implement wearable technology that continuously monitors vital signs and cardiac performance during training and competitions. This data can be integrated into predictive models to assess individual risks in real time.
- Personalized Risk Assessment: Utilize PCA plots and machine learning to create individualized profiles, identifying athletes’ specific risk factors for SCD. This enables tailored recommendations regarding training intensity, recovery protocols, and screenings.
- Interactive Visualization Tools: Develop user-friendly dashboards that display PCA diagrams in an interactive format, allowing clinicians to explore various datasets and understand the spatial distribution of patient clusters in a more engaging way.
- Collaborative Research Networks: Encourage partnerships between sports organizations, universities, and healthcare providers to share data on athlete health, which can enhance the development of robust predictive models and improve overall understanding of cardiac risks in sports.
- Educational Resources: Provide ongoing training and resources for clinicians on the latest advancements in sports cardiology, including interpretation of PCA data and implementing machine learning insights into clinical practice.

By adopting these cutting-edge strategies, clinicians can enhance their ability to assess, monitor, and manage the cardiac health of athletes, leading to improved outcomes and safer sports environments

## Conclusions

This study began with a dataset of 711 individuals who underwent screening to assess their physical fitness at a competitive level. We focused on variables correlated with SCD and initially selected twenty-six relevant variables from the literature. After refinement, we narrowed this down to eight core variables. Additionally, we incorporated other variables that we believed might be associated with the disease, resulting in a final set of forty-five variables. Consequently, we created two datasets with 711 entries each: one characterized by eight features ( **dataset_L_**) and the other by forty-five features ( **dataset_LE_**). We applied hierarchical clustering to both datasets using various metrics and combinations of similarity measures. The resulting clusterings were notably similar, allowing us to consistently reorganize the clusters. Specifically, we defined ten clusters for **dataset_L_** and eleven for **dataset_LE_**. We comprehensively interpreted all twenty-one clusters, emphasizing their potential medical significance. Following this, we visualized the data for both datasets using PCA. In most instances, elements of the clusters identified as associated with the disease were situated in regions distinctly separate from those considered healthy concerning SCD. We proposed using distance metrics from the clusters and PCA-based visualizations to assess risk in a diagnostic context. However, a significant limitation of these methods is the lack of a direct medical interpretation tied to specific formulas or indices. To address these challenges, future studies will focus on three key areas:

- *In-depth Analysis of Individual Clusters*: We will investigate the medical and statistical significance of each cluster identified in this research to enhance our understanding of their role in stratifying the risk of sudden cardiac death.
- *Development of Risk Equations*: We aim to derive mathematical equations that can define a risk index for sudden cardiac death based on the data from the clusters. This may involve using regression models, algorithms for feature selection such as genetic algorithms, and employing neural networks to optimize weights for predictive formulas.
- *Identification of Physically Meaningful Variables*: We will seek to find variables or data representations with explicit physical meaning that can help distinguish clusters in regions separate from those associated with healthy individuals. This could involve exploring dimensionality reduction techniques, advanced machine learning algorithms, or specialized feature selection methods, such as recursive feature elimination, principal component regression, or autoencoders, to derive latent features.

**Table 13:** Comprehensive list of the 151 variables recorded for 711 athletes undergoing pre-participation physical evaluation (PPPE). The variables are categorized into athlete data, family, physiological, and pathological history, clinical examination findings, resting and exertion electrocardiogram (ECG) results, spirometry, urinalysis, additional specialized tests, and final medical judgment.

Initial characteristics
ATHLETE DATA
(1) Number, (2) Birthdate, (3) Sex (F/M), (4) Practiced sport, (5) Sport class (A/B/C/D), (6) Visit type (First/Follow-up).
FAMILY HISTORY
(7) Sudden death (Y/N), (8) Heart disease (Grade I/II/Notes), (9) Hypertension (Y/N), (10) Diabetes DM1 (Grade I/II)/Diabetes DM2 (Grade I/II)/N, (11) Asthma (Grade I/II/NO), (12) Notes
PHYSIOLOGICAL HISTORY
(13) Pregnancy, (14) Menarche age, (15) Last menstruation, (16) Weight (Underweight/Overweight/Obese), (17) Smoking cigarettes/day, (18) Other
PATHOLOGICAL HISTORY
(19) Loss of consciousness (Y/N/Notes), (20) Epilepsy (Y/N/Notes), (21) Heart diseases, arrhythmias, etc. (Y/N/Notes), (22) Diabetes (DM1/DM2/N/Notes), (23) Asthma, (24) Allergies, (25) Pharmacological therapies, (26) Non-pharmacological therapies, (27) Notes
OTHER HISTORY
(28) Surgical interventions (Y/N/Notes), (29) Injuries (Y/N/Notes), (30) Information on Tetanus vaccine, (31) Mandatory Covid vaccine, (32) Disability (Y/N/Notes)
PHYSICAL EXAMINATION
Body composition: (33) Trophism, (34) Weight [kg], (35) Height [cm], (36) BMI (NUMERIC), (37) BMI. Resting blood pressure: (38) Systolic, (39) Diastolic, (40) Blood pressure. Blood group: (41) Type, (42) Rh factor, (43) Spine aligned; normal range of motion of joint appendages. Locomotor system: (44) Kyphosis, (45) Scoliosis, (46) Varus deformity, (47) Valgus deformity, (48) Flat foot, (49) Winged scapulae, (50) Pectus excavatum, (51) Pectus carinatum, (52) Other, (53) Chest and respiratory system (Normal chest expansion, FVT normally transmitted, (54) Normal breath sounds throughout the lung fields/N/Notes). Cardiovascular system (2 heart sounds, clear pauses, palpable and symmetric peripheral pulses/N/Notes). (55) Abdomen and genital organs (Notes). (56) Visual system: (57) Natural visual acuity right eye, (58) Natural visual acuity left eye, (59) Mandatory corrective lenses < 6 diopters in both eyes, (60) Corrected visual acuity right eye: (61) Corrected visual acuity left eye, (62) Stereoscopic visual field, (63) Color perception, (64) Mandatory lenses, (65) Whispered voice test, (66) Other, (67) Conclusions.
RESTING ELECTROCARDIOGRAM
(68) Mean heart rate, (69) PQ, (70) QT, (71) RS, (72) TS, (73) BS, (74) BPSV (Supraventricular extrasystole), (75) BPV (Ventricular extrasystole), (76) High voltage, (77) Repolarization abnormalities, (78) Early repolarization, (79) Respiratory sinus arrhythmia, (80) Atrial arrhythmia pacemaker, (81) Ventricular arrhythmia, (82) Right bundle branch block rsrv12, (83) Complete right bundle branch block, (84) Left bundle branch block, (85) Complete left bundle branch block, (86) AV block, (87) Right axis deviation, (88) Left axis deviation, (89) Anterior left hemiblock, (90) T wave negative (V3-4-5-6), (91) T wave negative (V1-V3), (92) Notched T wave, (93) Pathological Q waves, (94) Epsilon waves, (95) Short PR, (96) ST depression, (97) Atrial fibrillation, (98) Ventricular fibrillation, (99) Long QT syndrome, (100) Interventricular septum hypertrophy, (101) Lack of R wave progression in precordial leads, (102) Brugada Type 1, (103) Wolff-Parkinson-White syndrome
STRESS ELECTROCARDIOGRAM
(104) RS, (105) TS, (106) BS, (107) BPSV (Supraventricular extrasystole), (108) BPV (Ventricular extrasystole), (109) High voltage, (110) Repolarization abnormalities, (111) Early repolarization, (112) Respiratory sinus arrhythmia, (113) Atrial arrhythmia pacemaker, (114) Ventricular arrhythmia, (115) Right bundle branch block rsrv12, (116) Complete right bundle branch block, (117) Left bundle branch block, (118) Complete left bundle branch block, (119) AV block, (120) Right axis deviation, (121) Left axis deviation, (122) Anterior left hemiblock, (123) T wave negative (V3-4-5-6), (124) T wave negative (V1-V3), (125) Notched T wave, (126) Pathological Q waves, (127) Epsilon waves, (128) Short PR, (129) ST depression, (130) Atrial fibrillation, (131) Ventricular fibrillation, (132) Long QT syndrome, (133) Interventricular septum hypertrophy, (134) Lack of R wave progression in precordial leads, (135) Brugada Type 1, (136) Wolff-Parkinson-White syndrome
OTHER
Vital capacity: (137) Type, (138) Measured, (139) Theoretical. FEV1: (140) Measured, (141) Theoretical. Tiffeneau index: (142) Measured, (143) Theoretical. Maximum voluntary ventilation: (144) Measured, (145) Theoretical. (146) Spirometry notes. Urinalysis (147) Appearance, (148) pH, (149) Density, (150) Color, (151) Findings.
DIAGNOSTIC EVALUATION
(152) Additional specialized tests, (153) Other investigations, (154) Final medical judgment

By addressing these objectives, we aim to improve the interpretability and utility of this clustering-based approach, transitioning from abstract mathematical representations to practical diagnostic tools with clear physical and medical implications.

## Appendix

**Table 14:** Overview of features included in the datasets. This table summarizes the features included in the three datasets: Literature-based features and extra features (dataset_**LE**_) (45 variables), complete literature-based features (26 variables, and Literature-based features (dataset_**L**_) (8 variables), categorized into Risk and Physiological Factors, Resting ECG, and Stress ECG. Most features are binary (Y/N), except for Body Mass Index and Heart Rate.

Selected Characteristics
LITERATURE + EXTRA FEATURES (dataset_**LE**_) (All the 45 Data Matrix Columns)
RISK AND PHISIOLOGICAL FACTORS
(1) Class C Sport, (2) Family History of Heart Disease, (3) Personal History of Heart Disease, (4) Syncope, (5) Eutocic birth: Premature Delivery, (6) Caesarean section: Premature Delivery, (7) Pectus Excavatum, (8) Body Mass Index (numeric), (9) Body Mass Index, (10) Blood Pressure, (11) Heart Rate
RESTING ELECTROCARDIOGRAM
(12) Sinus Tachycardia, (13) Ventricular Extrasystole, (14) Atrial Arrhythmia, (15) Ventricular Arrhythmia, (16) Complete Right Bundle Branch Block, (17) Complete Left Bundle Branch Block, (18), Atrioventricular Block, (19) Right Axis Deviation, (20) Left Axis Deviation, (21) T-wave Inversion in the Lateral or Inferolateral Leads, (22) T-wave Inversion in the Anterior Leads, (23) Pathological Q waves (24) Ventricular pre-excitation, (25) ST-segment Depression, (26) Prolonged QTc, (27) Brugada Type 1 Pattern, (29) Wolff-Parkinson-White Syndrome
STRESS ELECTROCARDIOGRAM
(30) Sinus Tachycardia, (31) Ventricular Extrasystole, (32) Atrial Arrhythmia, (33) Ventricular Arrhythmi, (34) Complete Right Bundle Branch Block, (35) Complete Left Bundle Branch Block, (36) Atrioventricular Block, (37) Right Axis Deviation, (38) Left Axis Deviation, (38) T-wave Inversion in the Lateral or Inferolateral Leads, (39) T-wave Inversion in the Anterior Leads, (40) Pathologic Q waves, (41) Ventricular pre-excitation, (42) ST-segment Depression, (43) Prolonged QTc, (44) Brugada Type 1 Pattern, (45) Wolff-Parkinson-White Syndrome
LITERATURE FEATURES (Complete) (26 Columns)
RISK AND PHISIOLOGICAL FACTORS
(1) Class C Sport, (2) Family History of Heart Disease, (3) Personal History of Heart Disease, (5) Eutocic birth: Premature Delivery, (6) Caesarean section: Premature Delivery, (7) Pectus Excavatum, (8) Body Mass Index (numeric), (9) Body Mass Index, (10) Blood Pressure, (11) Heart Rate
RESTING ELECTROCARDIOGRAM
(12) Sinus Tachycardia, (14) Atrial Arrhythmia, (15) Ventricular Arrhythmia, (16) Complete Right Bundle Branch Block, (18), Atrioventricular Block, (19) Right Axis Deviation, (20) Left Axis Deviation
STRESS ELECTROCARDIOGRAM
(30) Sinus Tachycardia, (32) Atrial Arrhythmia, (33) Ventricular Arrhythmi, (34) Complete Right Bundle Branch Block, (36) Atrioventricular Block, (37) Right Axis Deviation, (38) Left Axis Deviation, (39) T-wave Inversion in the Anterior Leads, (44) Brugada Type 1 Pattern
LITERATURE FEATURES (*Dataset*_*L*_) (8 Columns)
RISK AND PHISIOLOGICAL FACTORS
(4) Syncope, (11) Heart Rate.
RESTING ELECTROCARDIOGRAM
(21) T-wave Inversion in the Lateral or Inferolateral Leads, (22) T-wave Inversion in the Anterior Leads, (24) Ventricular pre-excitation,
STRESS ELECTROCARDIOGRAM
(38) T-wave Inversion in the Lateral or Inferolateral Leads, (41) Ventricular pre-excitation, (43) Prolonged QTc
Note: all are Y/N variables except for (8) Body Mass Index (numeric), (9) Body Mass Index, and (11) Heart Rate.

**Table 15:** Eigenvalues and eigenvectors corresponding to the principal components. These values provide further insight into the structural patterns revealed by PCA, enhancing the understanding of the clustering results and their potential physical or clinical significance.

Eigenvalue	Eigenvector
**Literature**	
1.33948886	[-0.02574546 -0.21582806 -0.04223583 -0.05820833 0.69547565 -0.0164579 0.68039109 -0.0263454 ]
1.06033813	[-0.14748038 0.64559544 0.05575566 0.68059587 0.08135806 -0.05038276 0.16718894 -0.24088781]
**Literature + Extra**	
2.12520863	[ 1.66080203e-01 1.03668486e-01 4.34311644e-02 4.28818385e-03 2.61886992e-02 2.71806853e-02 -6.76431314e-02 5.98102972e-01 5.95472354e-01 8.31319959e-02 1.48738435e-01 7.65986995e-02 6.77626358e-21 -5.27449885e-02 -8.47032947e-22 -2.80764156e-02 -5.29395592e-23 1.28696767e-01 -1.71695788e-01 0.00000000e+00 4.66585785e-02 -7.57159580e-02 1.26217745e-28 -3.37059226e-02 5.54667824e-32 -4.81482486e-35 6.77084746e-36 -4.70197740e-38 2.29580194e-01 0.00000000e+00 -4.56044066e-02 0.00000000e+00 -3.50526060e-02 0.00000000e+00 1.58353649e-01 -1.60783652e-01 -4.54755848e-03 9.12079406e-02 -5.74175647e-02 0.00000000e+00 -9.63897395e-02 0.00000000e+00 1.41494470e-01 0.00000000e+00 0.00000000e+00]
1.82800413	4.50377478e-02 9.78295911e-02 -1.98681089e-02 -3.57731395e-02 7.60643334e-02 -4.68929749e-03 -2.73236940e-02 -1.26597069e-01 -1.27898297e-01 -3.44630542e-02 5.26104725e-01 4.57714074e-01 -0.00000000e+00 -9.05882094e-02 1.73472348e-18 -7.15433694e-02 -1.08420217e-19 -3.03742284e-01 -1.24884443e-01 -0.00000000e+00 -1.17703127e-02 3.94643917e-02 -0.00000000e+00 -7.20228128e-02 -2.01948392e-28 -0.00000000e+00 -7.39557099e-32 -0.00000000e+00 4.18877679e-01 -0.00000000e+00 -1.31083644e-02 -0.00000000e+00 3.08899815e-03 -0.00000000e+00 -3.07101052e-01 -1.16981610e-01 -4.41681151e-02 -8.37702447e-03 2.10408807e-03 -0.00000000e+00 2.90948652e-02 -0.00000000e+00 -2.13869812e-01 -0.00000000e+00 -0.00000000e+00]

## Supporting information

S1 Fig Graphical representation of the study. On the left, the study development: data from 711 athletes were analyzed using clustering and PCA, revealing that risk clusters occupy distinct regions. The proposed risk diagnostic tool is on the right: patient data are plotted on a region-based graph to determine whether the patient’s point falls within a high-risk area.
(TIFF)
