# Supplementary figures and images for: Identifying risk patterns for sudden cardiac death in athletes: A clustering and principal component analysis approach
Source: PLoS One. 2026 Jan 14;21(1):e0339377. doi: 10.1371/journal.pone.0339377 (PMC12803475; doi:10.1371/journal.pone.0339377)

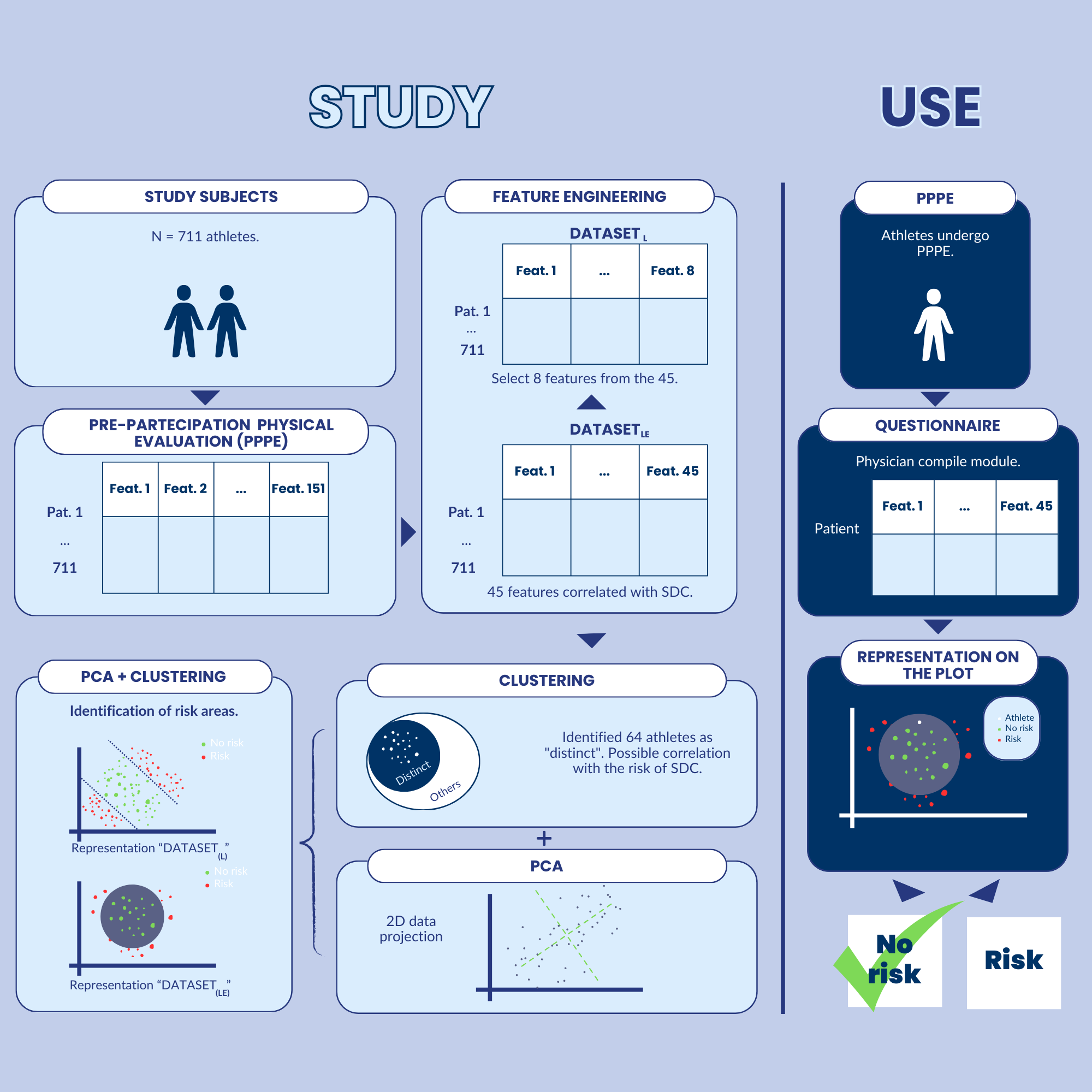

Supplement: S1 Fig — On the left, the study development: data from 711 athletes were analyzed using clustering and PCA, revealing that risk clusters occupy distinct regions. The proposed risk diagnostic tool is on the right: patient data are plotted on a region-based graph to determine whether the patient’s point falls within a high-risk area. (TIFF) [file pone.0339377.s001.tiff]
